# The impact of comprehensive public hospital reforms on the direct medical cost of inpatients with coronary heart disease

**DOI:** 10.3389/fpubh.2022.891186

**Published:** 2022-09-09

**Authors:** Liming Liu, Yue Xu, Jingfei Yu, Xiaowei Man, Yan Jiang, Liying Zhao, Wei Cheng

**Affiliations:** ^1^School of Management, Beijing University of Chinese Medicine, Beijing, China; ^2^Medical Insurance Office, Aerospace Center Hospital, Beijing, China; ^3^Shenzhen Beijing University of Traditional Chinese Medicine Research Institute, Shenzhen, China

**Keywords:** coronary heart disease, inpatients, direct medical expenses, comprehensive reform, interrupted time series analysis

## Abstract

**Objectives:**

To curb the unreasonable growth of medical expenses and reduce the burden of medical treatment, Beijing launched two rounds of comprehensive reform of public hospitals. In the two reforms, the addition of drugs and consumables was canceled successively. This study compared the changes in the direct medical cost of inpatients with coronary heart disease (CHD) in the three stages of two comprehensive public hospital reforms in Beijing and provides data support for health reform policies.

**Setting:**

CHD diagnosis and treatment data were extracted from the Hospital Information System (HIS) of 33 public hospitals. The total amount and composition of the direct medical expenses of CHD inpatients in the three stages were calculated. Interrupted time series analysis was used to study the instantaneous changes and trend changes in the three stages.

**Participants:**

The data were obtained from the HIS system of 33 public hospitals above the second level in Beijing. A total of 66,647 medical and diagnosis records and 24,371,139 charge detail records were included.

**Results:**

After the two reforms, the total cost for CHD inpatients with most clinical classifications and treatment methods decreased. The proportion of drug and consumable costs decreased significantly, whereas the proportion of medical consultation service costs increased. Drug-treated patients were mainly affected by the instantaneous reforms, percutaneous coronary intervention-treated patients were simultaneously affected by instantaneous and trending effects, and coronary artery bypass graft-treated patients were mainly affected by the reform trend.

**Conclusion:**

The overall change in the direct medical cost of CHD inpatients was consistent with the goal of the comprehensive medical reform of public hospitals in Beijing, which is “total control and structural adjustment.”

## Introduction

The rapid rise in health expenditures is an important issue in China's health field. From 2010 to 2017, China's total health expenditures increased by 12.78%, which was much higher than the gross domestic product (GDP) per capita growth rate of 8.06% (1). If this growth trend cannot be effectively curbed, the increase in health expenses may exceed the economic affordability of individuals, society, and the government (2, 3).

In the past, there was a 15% drug sale mark-up policy in public hospitals. Due to the drug markups, medical institutions or doctors might have exhibited “profit-seeking behaviors” (4). One of the reasons for the unreasonable increase in medical expenses was that hospitals received profit from drug markups to maintain normal operations. In 2017, the drug markup in public hospitals in China was completely canceled (5), which eliminated the relationship between doctors and drug revenue to a certain extent (6). Some studies found that the reform had a positive impact on doctors' medication behavior (7). But some studies found that although drug markups were abolished, doctors in public hospitals in China still had an incentive to prescribe more drugs (8). Other studies showed that although hospital drug revenue was reduced, it led to a rise in other revenue categories, such as diagnostics, laboratory tests, and medical consumables (9, 10).

Beijing launched two rounds of the comprehensive reform of public hospitals on 8 April 2017 and 15 June 2019. In the first round of reform, 15% of the drug markups (except for Chinese medicine decoction pieces), registration fees, and diagnosis and treatment fees were abolished, and medical service fees were set up. In addition, long-term prescription services for four types of chronic diseases, namely, hypertension, diabetes, coronary heart disease (CHD), and stroke, were launched. Long-term prescriptions for the four types of chronic diseases could be prescribed for up to 2 months (11). In the second round of reforms, consumables markups were canceled, and the volume-based procurement (VBP) of drugs and medical consumables was implemented (12). Both rounds of medical reform reduced the prices of inspection items and increased the prices of items that could reflect the value of doctors' labor, such as beds, nursing, traditional Chinese medicine, and surgery. The specific measures of the two rounds of reform are shown in Table 1.

**Table 1:** Reform measures at public hospitals in Beijing.

**Reform measures**	**The first round of reform**	**The second round of reform**
Zero mark-ups on sales of drugs and medical consumables	All public hospitals canceled the increase in drug sales (excluding Chinese herbal medicine pieces).	The medical consumables markup will be canceled, and medical institutions will charge the purchase price of medical consumables and will not sell them at a higher price.
Medical consultation service fees	Abolition of registration fees, medical treatment fees, and the establishment of medical consultation service fees. Medical consultation service fees are priced according to the institutional and physician-level gradient. For example, the consultation service fee for general physicians in primary medical and health institutions is 20 yuan, and the consultation service fee for well-known experts in tertiary hospitals is 100 yuan.	None
Adjustment of the price	Adjust the price and content of 435 medical services. Among them, the prices of examinations using large-scale equipment such as computed tomography (CT) and nuclear magnetic resonance decreased, and the price of some technical services such as surgery and acupuncture increased.	Reduce the price of some large-scale equipment inspection items. Increase the prices of five types of items, including traditional Chinese medicine, pathology, spirituality, rehabilitation, and surgery, which reflect the value of medical personnel's technical labor services.
Procurement of medicine and medical consumables	All medical institutions can negotiate, follow bids, and purchase on the Sunshine Procurement Platform.	Implement joint procurement of medical consumables and volume-based procurement of medicine.
Availability of medicine	The primary medical and health institutions are provided with drugs for the stable period of four chronic diseases including hypertension, diabetes, coronary heart disease, and cerebral apoplexy, and the prescription period of eligible patients in primary medical and health institutions is extended to 2 months.	None

CHD is a group of diseases that includes no symptoms, angina, myocardial infarction, ischemia cardiomyopathy, and sudden cardiac death (13). As one of the major forms of cardiovascular diseases (14), CHD is often accompanied by other diseases, such as hypertension, stroke, and diabetes (15). Therefore, CHD could impact not only locally in the heart, but also pulmonary function, whole-body skeletal muscle function, activity ability, psychological status, and others. Currently, with high morbidity and mortality, CHD has been the leading cause of death worldwide (13). The treatment of CHD is mainly divided into drug therapy and interventional therapy. In severe cases, surgical bypass surgery is considered (16). The average annual growth rate of hospitalization expenses far exceeds that of the GDP, which has exerted tremendous pressure on the economic burden of disease and the medical insurance funds of Chinese residents (17). As mentioned above, in this reform policy, CHD was one of the four types of chronic diseases for which medication could be prescribed for a long time.

Previous studies have been conducted on the impact of comprehensive reforms on the cost of all diseases and chronic diseases (11, 12, 18), but little attention has been paid to CHD. Therefore, the impact of reforms on CHD needs to be studied. This article is an important supplement to existing research.

Therefore, this study examined the changes in the cost for patients with CHD hospitalized before and after the two reforms, analyzed whether the results of the reform met the expected assumptions, and provided data support for my country to control the unreasonable growth of health costs, reduce irregular diagnoses and treatment behaviors, and adjust health policies.

### Research hypothesis

After implementing the two comprehensive reforms in public hospitals, the total direct medical expenses of CHD inpatients remained unchanged or decreased, and the structure of direct medical expenses was optimized.

## Methods

### Data sources

Data were obtained from the Hospital Information System (HIS) of 33 public hospitals in Beijing, which ensures the authenticity, accuracy, and completeness of the data. Data were obtained from the Medical Record Table, the Diagnosis Record Table, and the Charge Details Table. The Medical Record Table mainly includes basic information such as the patient's admission date, discharge date, and hospital name. The Diagnosis Record Table includes information related to the patient's condition and treatment, including main diagnosis, secondary diagnosis, main surgery/operation, and secondary surgery/operation. The Charge Details Table includes detailed information about the patient's diagnosis and treatment, medical service items, the unit price of medical service items, and the quantity of medical service items used.

### Data processing

According to the research purpose of this article, the data of patients whose first three main diagnosis ICD-10 code digits ranged from I20 to I25 and whose discharge dates were between 1 January 2016 and 31 December 2019 were extracted. Then, the data were cleaned to delete duplicate data records, missing key field records, and abnormal data. After that, records with the primary diagnosis of CHD were included, and records with any secondary diagnosis were excluded. Finally, a total of 51,951 medical records were included.

Since there are many clinical types of CHD, this study selected the clinical types with a large number of medical records for analysis. In different treatment methods and different reform stages, unstable angina pectoris (I20.0), acute subendocardial myocardial infarction (I21.4), and arteriosclerotic heart disease (I25.1) had a considerable number of patient records. To reduce the randomness of the data, we choose these three clinical classifications for this research. At the same time, due to the large differences in costs between the various treatment methods, treatments were divided into three groups, namely medical treatments, percutaneous coronary intervention (PCI), and coronary artery bypass grafting (CABG).

In addition, according to the time points of the implementation of the two reforms, the included patient records were divided into three stages:

① The period before the implementation of the reform was classified as the pre-reform stage, with patient discharge dates between 1 January 2016 and 7 April 2017.
② The period after the implementation of the first reform was classified as the first reform stage, with admission dates on or after 8 April 2017 and a discharge date before 14 June 2019.
③ The period after the second reform trial was classified as the second reform stage. The admission date was on or after 15 June 2019, and the discharge date was before 31 December 2019.

### Descriptive statistical analysis

Descriptive statistical analysis of the data was carried out using Excel software. The total cost for CHD patients and the proportion of various detailed costs in the three stages were counted separately, including the cost of medicine, the cost of consumables, the cost of inspections, and the cost of laboratory and medical consultation services. The content of each cost is shown in Table 2. The cost of medical consultation services (19) was the sum of the cost of registration, bed, examination, treatment, surgery, pharmacy services, and nursing care. Since these types of expenses are designed to reflect the value of the technical services of the medical staff, they were summed as the medical consultation service costs in the results.

**Table 2:** Fee explanation list.

**Fee name**	**Meaning**
Cost of medical consultation services	Refers to the expenses incurred from medical-technical services provided by medical personnel, which are the total expenses of the patient's hospitalization expenses after excluding examination expenses, laboratory expenses, drug expenses, and consumables expenses.
Cost of inspection	Refers to the relevant fees for examining, diagnosing, and treating patients using CT, MRI, and other equipment.
Cost of laboratory	Refers to the costs related to laboratory tests performed by patients during hospitalization, including routine blood and urine tests.
Cost of medicine	Refers to the cost of medicine used by the patients for the treatment of diseases, including Western medicine and traditional Chinese medicine.
Cost of consumables	Refers to the cost of separately chargeable medical consumables consumed by a patient during hospitalization.

### Interrupted time series analysis

Interrupted time series analysis (ITSA) is a quasi-experimental research design used to evaluate the effects of interventions (20). It measures the instantaneous changes and trend changes from the intervention by observing data at multiple time points before and after the intervention. This method has been applied to the field of policy evaluation (21). The segmented regression model is commonly used in ITSA. Through segmented regression model analysis, the trend in the actual results after the intervention and the natural trend before the intervention were compared and tested to evaluate the intervention effect (22). The ITSA model principle was detailed in a previous report (23).

The monthly data were summarized, and then, a piecewise regression model of ITSA was established. Through ITSA, the instantaneous changes and trend changes in the monthly average cost for patients with different treatment methods and clinical types of CHD before and after the two reforms were discussed to explore whether the changes in CHD cost changes were mainly affected by instantaneous effects or trends.

## Results

The basic characteristics of the patients in the three reform stages are shown in Table 3. The proportion of male patients was slightly higher than that of female patients, the average age was about 66 years old, and the highest proportion of patients aged 60–69 was about 33%. The average length of hospitalization was about 7 days, most patients had an ICD-10 code of I25.1, and the proportion of patients receiving drug treatment was more than in patients treated with PCI or CABG.

**Table 3:** Characteristics of patients in the three reform stages.

	**The first stage**	**The second stage**	**The third stage**
*N*	16,511	29,756	5,684
Gender (*N*, %)			
Male	9,892 (59.91)	16,891 (56.77)	3,119 (54.87)
Female	6,619 (40.09)	12,865 (43.23)	2,565 (45.13)
Age, years (mean, SD)	65.31 (11.91)	66.21 (11.97)	66.30 (12.22)
Age group (*N*, %)			
≤ 39	192 (1.16)	342 (1.15)	98 (1.72)
40–49	1,269 (7.69)	2,010 (6.75)	343 (6.03)
50–59	3,875 (23.47)	6,307 (21.20)	1,200 (21.11)
60–69	5,364 (32.49)	9,880 (33.20)	1,845 (32.46)
70–79	3,540 (21.44)	6,555 (22.03)	1,272 (22.38)
≥80	2,271 (13.75)	4,662 (15.67)	926 (16.29)
Hospital days (mean,SD)	7.01 (5.11)	7.79 (5.70)	7.67 (5.00)
Hospital days grouping (*N*, %)			
≤ 7	11,414 (69.13)	17,861 (60.02)	3,454 (60.77)
>7	5,097 (30.87)	11,895 (39.98)	2,230 (39.23)
ICD-10 code (*N*, %)			
I20.0	4,545 (27.53)	12,821 (43.09)	2,907 (51.14)
I21.4	1,398 (8.47)	2,252 (7.57)	384 (6.76)
I25.1	10,568 (64.01)	14,683 (49.34)	2,393 (42.10)
Treatment (*N*, %)			
Medical treatment	11,525 (69.80)	22,024 (74.02)	4,153 (73.06)
PCI	4,689 (28.40)	6,609 (22.21)	1,176 (20.69)
CABG	297 (1.80)	1,123 (3.77)	355 (6.25)

1. SD refers to standard deviation. 2. I20.0 refers to unstable angina pectoris; I21.4 refers to acute subendocardial myocardial infarction; I25.1 refers to arteriosclerotic heart disease. 3. PCI refers to percutaneous coronary intervention; CABG refers to coronary artery bypass grafting.

Table 4 shows the average total cost for patients with ICD codes CHDI20.0, I21.4, and I25.1 treated with medication, PCI, and CABG.

**Table 4:** Average total cost of inpatients with coronary heart disease in three stages of different treatment methods and clinical types [1,000 RMB (1,000 dollars)].

**Treatment**	**ICD-10** **code**	**Pre-reform** **stage**	**First reform** **stage**	**Second reform** **stage**	**Growth** **rate (%)**
Medical treatment	I20.0	5.73 (1.55)	5.77 (1.68)	5.18 (1.46)	−9.62
	I21.4	9.18 (2.48)	10.27 (3)	8.5 (2.4)	−7.37
	I25.1	6.92 (1.87)	6.65 (1.94)	6.45 (1.82)	−6.78
PCI	I20.0	32.17 (8.7)	31 (9.05)	28.16 (7.93)	−12.46
	I21.4	36.13 (9.77)	34.91 (10.19)	30.98 (8.73)	−14.26
	I25.1	32.76 (8.86)	32.9 (9.6)	35.43 (9.98)	8.15
CABG	I20.0	70.13 (18.97)	56.32 (16.44)	64.31 (18.12)	−8.30
	I21.4	79.05 (21.38)	72.37 (21.12)	99.8 (28.12)	26.25
	I25.1	60.55 (16.38)	59.71 (17.43)	62.33 (17.56)	2.93

1. PCI refers to percutaneous coronary intervention; CABG refers to coronary artery bypass grafting. 2. I20.0 refers to unstable angina pectoris; I21.4 refers to acute subendocardial myocardial infarction, I25.1 refers to arteriosclerotic heart disease.

In medical treatment, the total cost of the patients with the three clinical types of CHD increased from the first phase of the reform and decreased in the second phase of the reform. After the two reforms, the overall cost for patients with ICD codes of I20.0 decreased by 9.62% compared to before the medical reforms, and the cost for patients with I21.4 and I25.1 ICD codes increased by 7.37 and 6.78%, respectively.

In PCI treatment, the total cost for patients with the three clinical types of CHD increased in the first phase of the reform, and the cost for patients with ICD codes I20.0 and I21.4 decreased in the second phase of the reform, while the cost for patients I25.1 increased. After the two reforms, the overall cost for patients with I20.0 and I21.4 ICD codes decreased by 12.46 and 14.26%, respectively, and the cost for patients with I25.1 ICD codes increased by 8.15%.

In CABG treatment, the total cost for patients with ICD codes I20.0 and I21.4 decreased from the first phase of the reform, while the cost for patients with I25.1 ICD codes increased, and the cost for patients with the three clinical types of CHD increased in the second phase of the reform. After the two reforms, the overall cost for patients with ICD codes of I20.0 decreased by 8.30% compared to before the medical reforms, and the cost for patients with I21.4 and I25.1 ICD codes increased by 26.25 and 2.93%, respectively.

**Figure 1:**
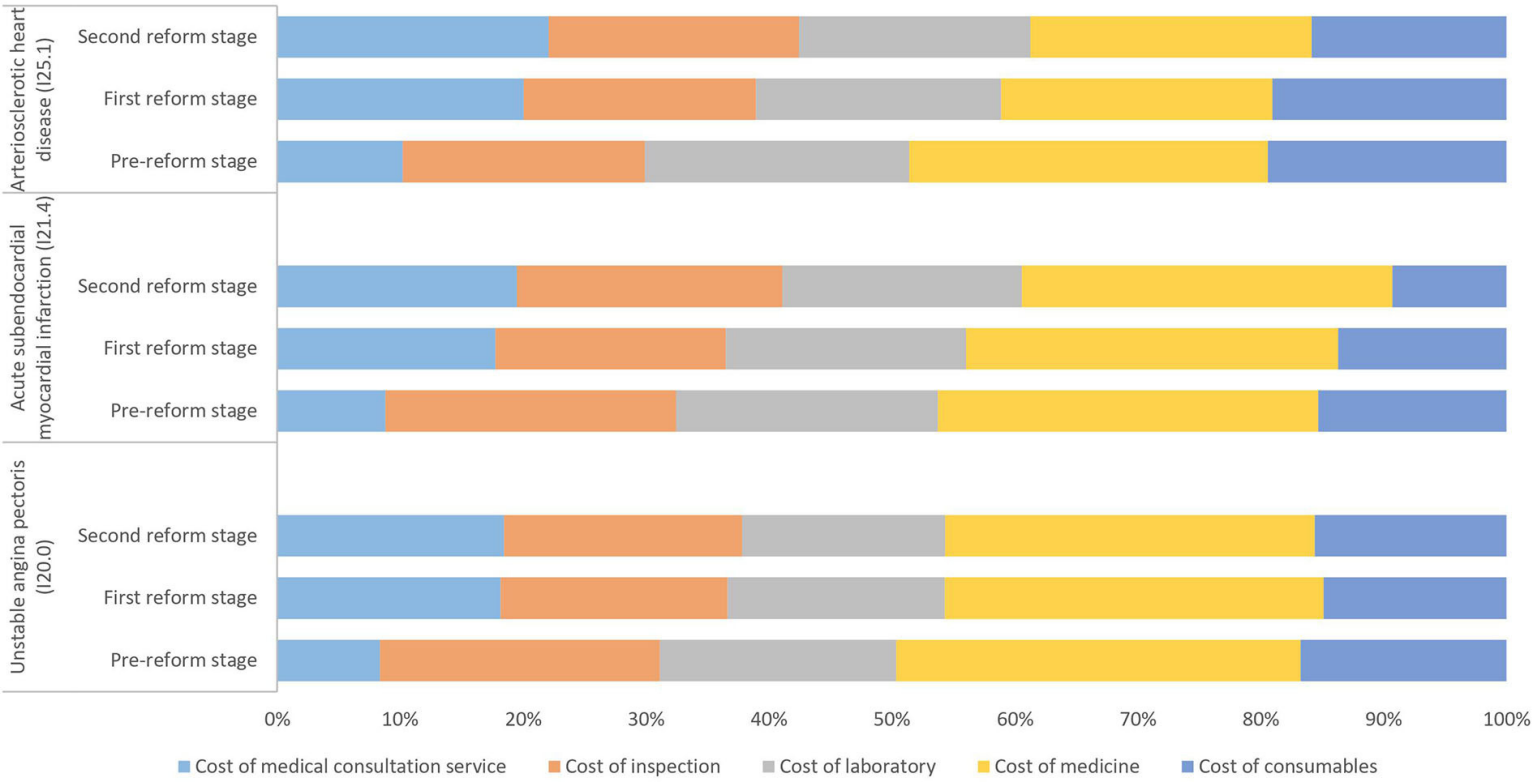
Composition of the total cost of inpatients with different clinical types of coronary heart disease treated by medical treatment in the three reform stages (%).

Figure 1 shows the composition of the average total cost for the medical of treatment inpatients with three clinical types of CHD. From the perspective of the cost structure, the cost of medicine accounted for a relatively high proportion of the cost of CHD patients treated with medications. After the two reforms, the proportion of the cost of medicine dropped. Among them, the proportion of the cost of medicine in patients with I25.1 ICD codes dropped the most, which was 6.28%. The proportion of the cost of medical consultation services increased by more than 10%; the proportion of the cost of consumables decreased, of which the proportion of consumables for patients with ICD codes of I21.4 and I25.1 dropped by 6 and 4%, respectively, more than the decrease of 1% in patients with ICD codes of I20.0. The proportion of the cost of inspections and laboratory tests decreased by about 2–3%.

**Figure 2:**
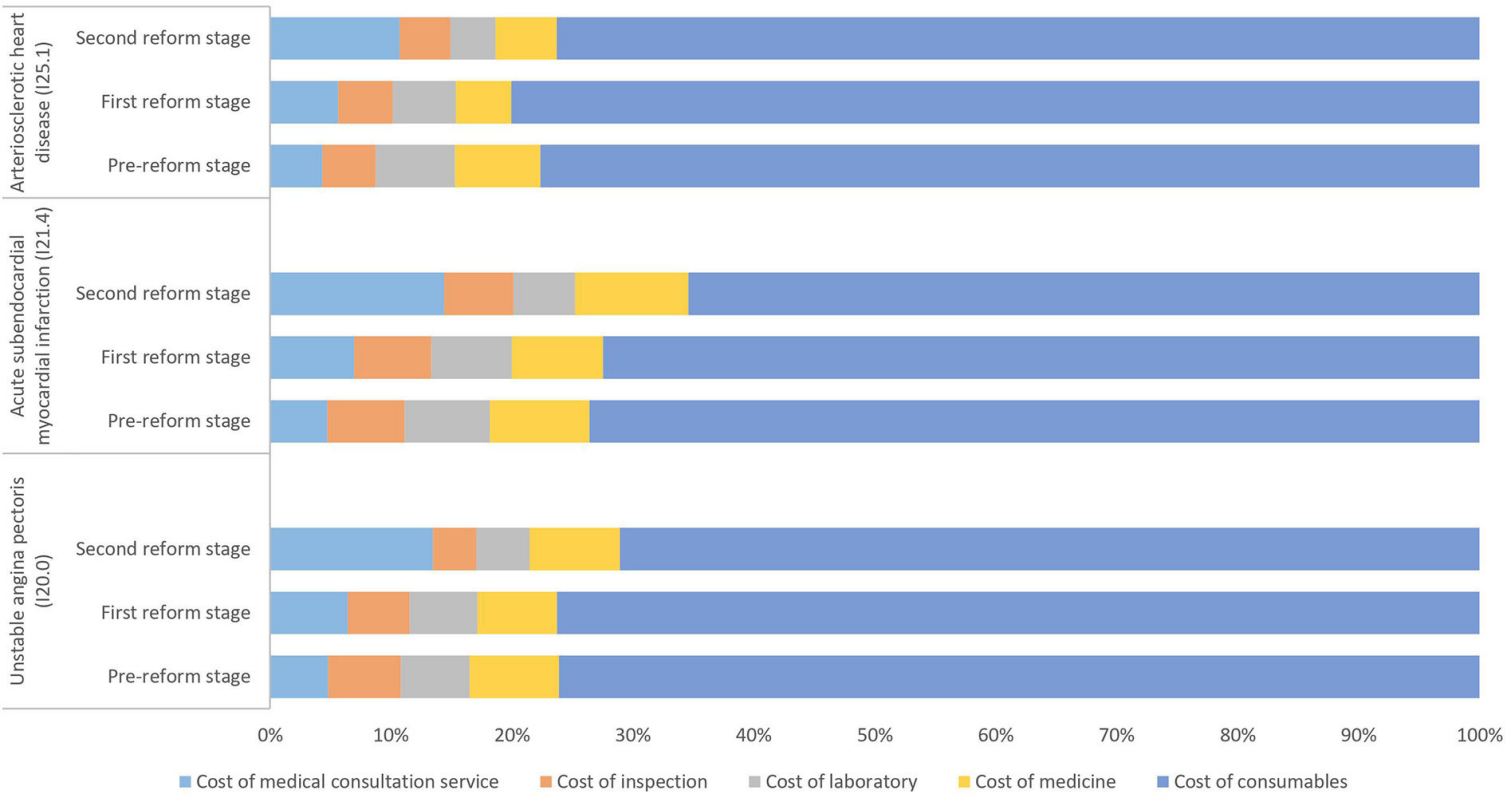
Composition of the total cost of inpatients with different clinical types of coronary heart disease treated with percutaneous coronary intervention (PCI) in the three reform stages (%).

Figure 2 shows the composition of the average total cost of inpatients with three clinical types of CHD treated with PCI. From the perspective of cost composition, the proportion of the cost of consumables for patients with CHD treated by PCI was relatively high. After the two reforms, the proportion of the cost of consumables decreased, especially in patients with ICD codes I20.0 and I21.4, which decreased by 5 and 8%, respectively. The proportion of the cost of medical consultation services increased by about 6–9%. The proportion of the cost of medicine, inspections, and laboratory tests did not change much, at < 3%.

**Figure 3:**
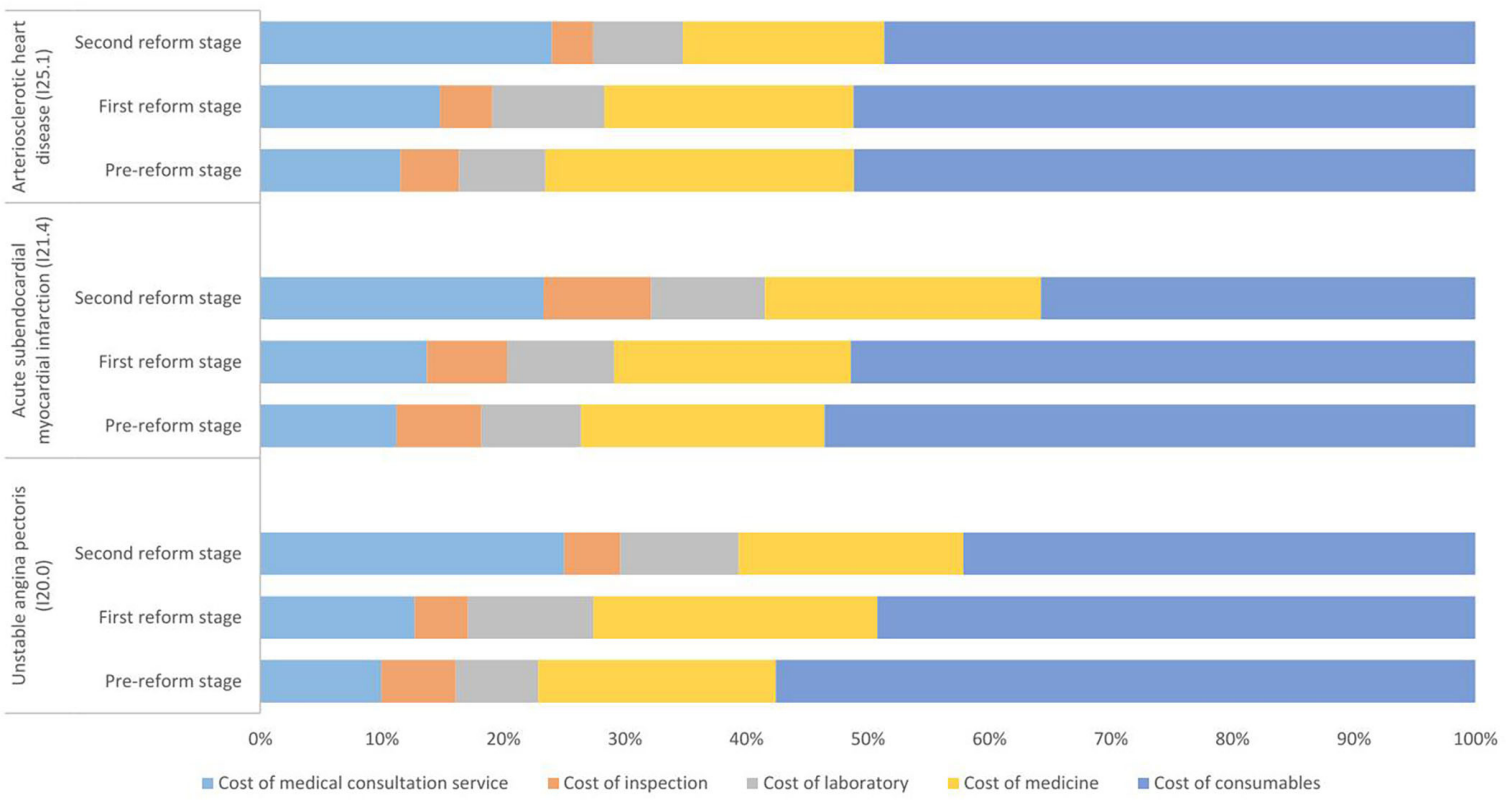
Composition of the total cost of inpatients with different clinical types of coronary heart disease treated with coronary artery bypass grafting (CABG) in three reform stages (%).

Figure 3 shows the composition of the average total cost of inpatients with the three clinical types of CHD who underwent CABG treatment. From the perspective of cost composition, the proportion of the cost of consumables for CHD patients treated by CABG was relatively high. After the two reforms, the proportion of the cost of consumables decreased, especially for patients with ICD codes of I20.0 and I21.4, which dropped by more than 15%, and the proportion of medical consultation service expenditure increased by 12–15%. The proportion of the cost of medicine in patients with ICD codes of I25.1 decreased by 8.84%, and the proportion of the cost of inspections and laboratory tests changed little, at < 3%.

**Figure 4:**
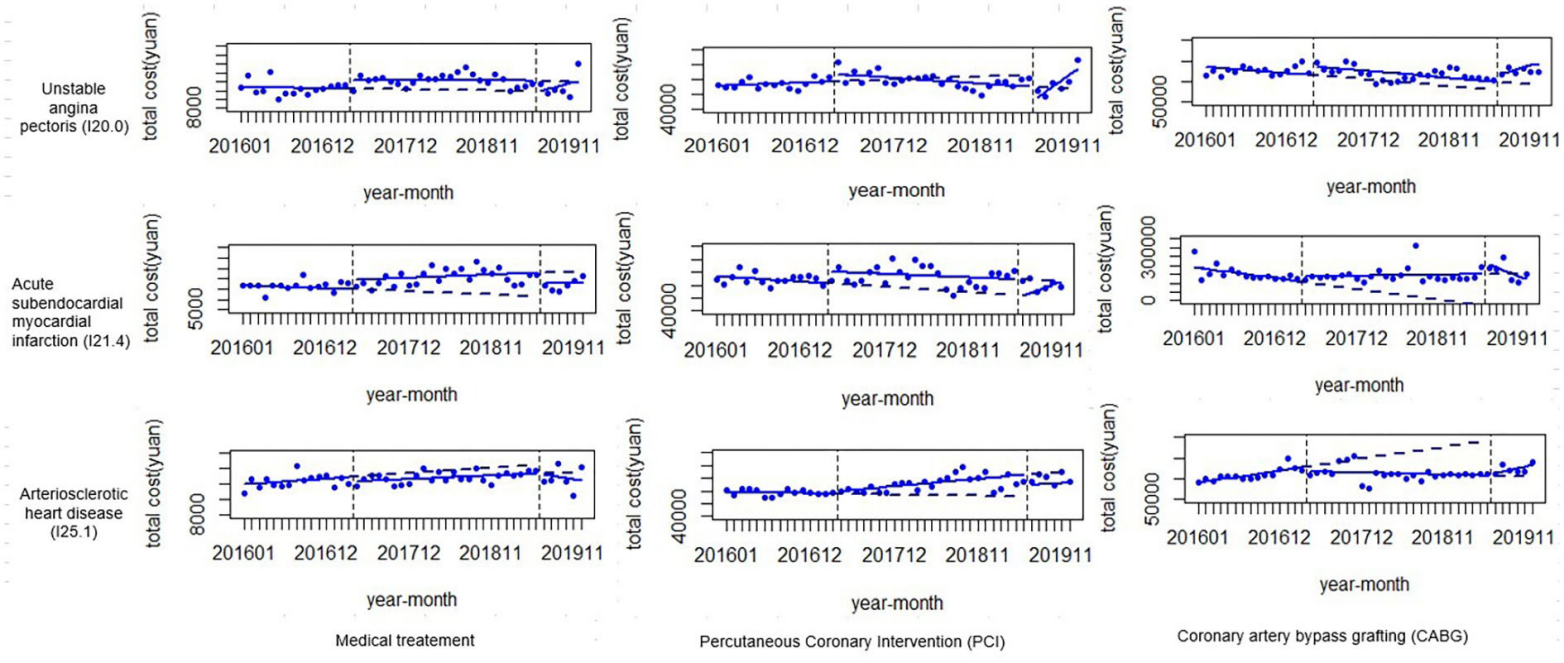
ITSA of the average total cost of inpatients with coronary heart disease with different treatment methods and clinical types.

Figure 4 shows the instantaneous changes and trend changes in the total cost of CHD inpatients with different clinical types and treatment methods after the two reforms. The ITSA results are shown in Table 5.

**Table 5:** ITSA regression results of hospital costs in CHD patients with different treatment methods and clinical types.

**Treatment**	**ICD-10** **code**	**The first round of reform**			**The second round of reform**	
		**Pre-reform** **trends**	**Reform instant** **change**	**Post-reform** **trend change**	**Reform instant** **change**	**Post-reform** **trend change**
Medical treatment	I20.0	−15.49	1,068.81	10.7	−1,587.77	272.58
		(−0.32)	(2.08)*	−0.2	(−1.96)^·^	−1.41
	I21.4	−126.25	4,437.82	270.7	−5,118.63	−128.34
		(−0.92)	(3.46)**	(2.07)*	(−2.52)*	(−0.27)
	I25.1	66.48	−594.53	−25.36	117.04	−211.57
		(2.73)**	(−2.36)*	(−1.03)	−0.18	(−1.26)
PCI	I20.0	200.35	5,228.68	−544.15	−10,777.18	4,126.77
		−0.77	(1.88)^·^	(−1.88)^·^	(−2.55)*	(4.07)***
	I21.4	−383.95	8,918.85	143.52	−14,309.34	2,246.69
		(−3.61)***	(19.09)***	−1.122	(−7.30)***	(3.49)**
	I25.1	−86.55	1,411.11	622.94	−8,195.19	−256.54
		(−0.41)	−0.66	(2.94)**	(−2.05)*	(−0.26)
CABG	I20.0	−1,386.05	20,770.8	−103.49	14,354.57	6,561.65
		(−1.62)	(2.45)*	(−0.12)	−1.33	(2.30)*
	I21.4	−5,427.76	22,338.28	5,981.65	75,031.14	−17,520.13
		(−2.91)**	−1.11	(2.99)**	(2.12)*	(−2.05)*
	I25.1	2,613.08	−9,633.27	−3,044.34	2,987.14	4,292.28
		(2.55)*	(−0.89)	(−2.62)*	−0.19	−1.14

1. The values outside the brackets are the amount of change, and the values in the brackets are the t-values. 2. ***, **, *, and · are significant at 0.001, 0.01, 0.05, and 0.1, respectively. If not marked, the p-value is > 0.1. 3. I20.0 refers to unstable angina pectoris; I21.4 refers to acute subendocardial myocardial infarction; I25.1 refers to arteriosclerotic heart disease. 4. PCI refers to percutaneous coronary intervention; CABG refers to coronary artery bypass grafting.

In patients with medical treatment, the cost for patients with I20.0 ICD codes rose instantaneously after the first reform and dropped instantaneously after the second reform, whereas the cost for patients with I21.4 ICD codes rose instantaneously after the first reform and showed an upward trend. After the second reform, it showed an instantaneous decline and patients with I25.1 ICD codes dropped instantaneously after the first reform.

In those with PCI treatment, the cost for patients with I20.0 ICD codes rose instantaneously after the first reform and then showed a downward trend, fell instantaneously after the second reform, and then showed an upward trend. The cost for patients with I21.4 ICD codes rose instantaneously after the first reform, and after the second reform, it dropped instantaneously and then showed an upward trend. Patients with I25.1 ICD codes showed an upward trend after the first reform and dropped instantaneously after the second reform.

In those who underwent CABG treatment, the cost for patients with I20.0 ICD codes rose instantaneously after the first reform and showed an upward trend after the second reform. The cost for patients with I21.4 ICD codes showed an upward trend after the first reform and, immediately after the second reform, then showed a downward trend. The cost for patients with I25.1 ICD codes showed a downward trend after the first reform.

## Discussion

This study observed the changes in the direct medical expenses of inpatients with different treatment methods and disease types after the two reforms through the average total costs at different stages of the reform. The results showed that after two reforms, the cost for the three types of patients with medical treatment decreased, especially in patients with I20.0 ICD codes. The cost for PCI-treated patients with ICD codes I20.0 and I21.4 decreased, and the cost for patients with I25.1 ICD codes increased. The cost for patients with ICD codes of I20.0 treated with CABG decreased, and the cost for patients with ICD codes of I21.4 and I25.1 increased especially in patients with ICD codes of I21.4. Overall, the cost for most types of CHD patients decreased, especially for patients treated with drugs, but the cost for some types of PCI and CABG patients showed a rising trend, which may have been due to the reform policy requirements. It is necessary to fully reflect the value of the technical labor of the medical staff. Compared to medical treatment, PCI and CABG are valuable adjuncts rather than an alternative to medical therapy (24), which reflects the greater value of technical labor, so the cost may be greater. In short, since the reforms, medical expenses have been effectively controlled. However, the rising expenses need continuous attention in future hospitalization expense management and control to continue to control the cost burden of CHD patients.

This study also observed changes in the composition of direct medical expenses for CHD patients after the reforms. First, the cost of medical consultation services included all costs that could reflect the technical value of the medical staff (12). The higher the proportion of medical consultation service costs, the better the labor value of the medical staff. The results of the study showed that the proportion of the cost of medical consultation services for the medical treatment of CHD patients rose about 20%, indicating the labor value of the medical staff after the reforms. The proportion of the cost of medical consultation services involving PCI and CABG treatment of CHD patients accounted for about 14 and 24%, respectively, which was more than before the reform, and its proportion was lower than that during medical treatment, mainly because the current patients treated by these two treatment methods used mostly consumables and medicine. However, considering the operating difficulty and medical risks of these two treatment methods, the value of medical staff labor should be more reflected, and the composition of the cost needs to be further optimized. In future, it is necessary to scientifically quantify the technical value of medical personnel labor based on the level of economic development in the region, reflecting the difficulty and risk of medical operations. Through the linkage reform of medicine, medical insurance, and medical care (25), the value of medical personnel labor is reflected while ensuring that the patient's disease burden does not increase.

In addition, the proportion of the cost of medicine and consumables reflects the burden of the patients' costs of medicine and consumables, respectively. To reduce the cost of drugs and consumables, the markup of drugs and consumables was eliminated in the first reform and the second reform, respectively. The results of the study showed that drug costs accounted for the highest proportion of the medical treatment of patients. After the reform, the proportion of the drug costs declined, in agreement with reform expectations, and the burden of drug costs has been effectively controlled. In addition, the cost of consumables accounted for the highest proportion of the cost of PCI and CABG patient treatments. After the reform, the proportion of the cost of consumables dropped significantly, which is also consistent with the reform goals, and the burden of consumables has also been controlled. However, the cost of consumables remained the main cost of the PCI- and CABG-treated patients. In future, with the advancement of the VBP of drugs and medical consumables, the price will continue to fall, and the economic burden will continue to be controlled (26).

From the perspective of changes in the total amount and the composition of comprehensive costs, the overall cost of CHD patients after the reform has not changed much, and the proportion of medical consultation service expenses has increased, whereas the proportion of medicine and consumables has decreased, realizing the “translation” effect of the reform on expenses. The cost has also shifted from medicine and consumables to medical consultation services, which can better reflect the value of technical medical staff labor in the diagnosis and treatment processes. The cost structure has been optimized, which is conducive to the rationalization of diagnosis and treatment behaviors, thereby enhancing the value of medical consultation services. Overall, the results of this study are consistent with the research hypothesis. Previous studies evaluated the two rounds of public hospital reforms in Beijing and found that both rounds of reform contained the growth rate of medical expenditures, and hospital revenue structures were reshaped and optimized (12), which are consistent with the results of this study.

This study also analyzed the instantaneous changes and trend changes in the direct medical expenses of CHD patients through ITSA. Overall, after the reform, medically treated patients had a transient effect, CABG-treated patients had a trend effect, and PCI-treated patients had both transient and trend effects. From a clinical point of view, drug treatment is the basis of all treatments, so only the patient's cost of drug treatment has an immediate change, indicating that the policy of reforming and canceling drug markups had an overall impact on the cost level, but did not change the trend of cost changes. PCI is carried out on the basis of drug therapy, which includes the technical difficulty of surgery in addition to drug therapy, so there are both instantaneous and trend changes. The absence of instantaneous changes in the cost of CABG may be that bypass therapy is more affected by technology and less affected by drugs (27).

In addition, the total cost for patients with the I20.0 ICD codes mentioned above for medical treatment decreased overall compared to before the reforms, and the ITSA results found that the cost of this type of patient only changed instantaneously, and the trend change was not significant. After the first reform, the cost for patients with I20.0 ICD codes rose instantaneously and then dropped instantaneously after the second reform. Therefore, it was comprehensively judged that the cost was mainly due to the instantaneous impact of the second reform.

In addition, as mentioned above, the total cost for patients with I20.0 and I21.4 ICD codes for PCI treatment decreased, and the cost for patients with I25.1 ICD codes increased. The ITSA results showed that after the first reform, the cost for patients with I20.0 and I21.4 ICD codes rose instantaneously, and the cost for patients with I20.0 ICD codes showed a downward trend. After the second reform, the cost for patients with I20.0 and I21.4 ICD codes dropped instantaneously and then showed an upward trend. Therefore, it can be inferred that the decline in the costs for patients with I20.0 ICD codes was not only affected by the trend of the first reform, but also by the instantaneous impact of the second reform, and the decline in the costs for patients with I21.4 ICD codes was mainly due to the instantaneous effects from the second reform. In addition, patients with I25.1 ICD codes showed an upward trend after the first reform and dropped instantaneously after the second reform, which could indicate that the increase in the cost for patients with I25.1 ICD codes was mainly affected by the trend change of the first reform.

Finally, the above discussion mentioned increases in the costs for patients with I21.4 and I25.1 ICD codes treated with CABG. The ITSA results showed that in CABG treatment, the cost for patients with I21.4 ICD codes showed an upward trend after the first reform, rose instantaneously after the second reform, and then showed a downward trend. Therefore, the increase in the cost for patients with I21.4 ICD codes was mainly affected by the change in the trend from the first reform and the instantaneous change from the second reform. In addition, patients with I25.1 ICD codes showed a downward trend after the first reform, but the costs increased overall, indicating that the changes in the trend after the reform did not significantly change the costs.

### Strengthens and limitations

First, previous studies only explained the effect in overall diseases and chronic diseases. This study additionally confirmed the effectiveness of the comprehensive reforms in CHD, that is, the role of reform in CHD was consistent with the overall change in previous studies.

Second, since there were large differences in costs between the various treatment methods, we specifically divided them into three groups, namely medical treatment, PCI, and CABG, which enriched the results.

Third, this study had advantages in data collection, and the sample was highly representative, but it only represented the situation of medical reform in Beijing. Due to the specificity of Beijing in China, the extrapolation of the study results is limited.

## Conclusion

This study observed the changes in the total amount and composition of the direct medical expenses of CHD patients after the two comprehensive reforms of public hospitals in Beijing, as well as the instantaneous and trend changes. Finally, we found that the total cost of inpatients with coronary heart disease agreed with the goal of “total control and structural adjustment” in Beijing's comprehensive medical reform. After the reform, the total cost was effectively controlled and the cost structure was optimized. The proportion of medical consultation service costs greatly increased under the guidance of comprehensive reform policy, reflecting the improvement in the value of medical staff labor. At the same time, the proportion of the cost of medicine and consumables decreased, and the burden in CHD patients was reduced. On the whole, the results are consistent with the expected effect of the reform policy.

## Data availability statement

The original contributions presented in the study are included in the article/supplementary material, further inquiries can be directed to the corresponding author.

## Author contributions

WC conceived and designed the experiments. XM, YJ, LZ, and YX performed the experiments. LL and YX analyzed the data, contributed reagents, materials, and analysis tools. LL and JY wrote the article. All authors contributed to the article, reviewed the manuscript and approved the submitted version.

## Funding

Funding was obtained from 2021 Accounting of Beijing's Total Recurring Health Expenditure Based on SHA2011 and the fund project only provided basic data support for this study.

## Conflict of interest

The authors declare that the research was conducted in the absence of any commercial or financial relationships that could be construed as a potential conflict of interest.

## Publisher's note

All claims expressed in this article are solely those of the authors and do not necessarily represent those of their affiliated organizations, or those of the publisher, the editors and the reviewers. Any product that may be evaluated in this article, or claim that may be made by its manufacturer, is not guaranteed or endorsed by the publisher.
